# Frequent Transient Hepatitis C viremia without Seroconversion among Healthcare Workers in Cairo, Egypt

**DOI:** 10.1371/journal.pone.0057835

**Published:** 2013-02-28

**Authors:** Aline Munier, Diaa Marzouk, Florence Abravanel, Mai El-Daly, Sylvia Taylor, Rasha Mamdouh, Waleed Salah Eldin, Hanan Ezz El-Arab, Dalia Gaber Sos, Mohamed Momen, Omar Okasha, Lenaig Le Fouler, Mostafa El-Hosini, Jacques Izopet, Mona Rafik, Matthew Albert, Mohamed Abdel-Hamid, Mostafa Kamal Mohamed, Elisabeth Delarocque-Astagneau, Arnaud Fontanet

**Affiliations:** 1 Emerging Diseases Epidemiology Unit, Institut Pasteur, Paris, France; 2 Department of Community, Environmental and Occupational Medicine, Faculty of Medicine, Ain Shams University, Cairo, Egypt; 3 INSERM, U1043, Centre de Physiopathologie de Toulouse Purpan, Toulouse, France; 4 CHU Toulouse, Hôpital Purpan, Laboratoire de virologie, Institut fédératif de biologie de Purpan, Toulouse, France; 5 Viral Hepatitis Research Laboratory, National Hepatology and Tropical Medicine Research Institute, Cairo, Egypt; 6 National Liver Institute, Minoufiya University, Minoufiya, Egypt; 7 Faculty of Medicine, Aim Shams University, Cairo, Egypt; 8 Laboratory of Dendritic Cell Immunobiology, Institut Pasteur, Paris, France; 9 INSERM U818, Paris, France; 10 Department of Microbiology, Minia Faculty of Medicine, Minia, Egypt; 11 Conservatoire National des Arts et Métiers, Chaire Santé et Développement, Paris, France; Duke University, United States of America

## Abstract

**Backgrounds:**

With 10% of the general population aged 15–59 years chronically infected with hepatitis C virus (HCV), Egypt is the country with the highest HCV prevalence worldwide. Healthcare workers (HCWs) are therefore at particularly high risk of HCV infection. Our aim was to study HCV infection risk after occupational blood exposure among HCWs in Cairo.

**Methodology/Principal Findings:**

The study was conducted in 2008–2010 at Ain Shams University Hospital, Cairo. HCWs reporting an occupational blood exposure at screening, having neither anti-HCV antibodies (anti-HCV) nor HCV RNA, and exposed to a HCV RNA positive patient, were enrolled in a 6-month prospective cohort with follow-up visits at weeks 2, 4, 8, 12 and 24. During follow-up, anti-HCV, HCV RNA and ALT were tested. Among 597 HCWs who reported a blood exposure, anti-HCV prevalence at screening was 7.2%, not different from that of the general population of Cairo after age-standardization (11.6% and 10.4% respectively, p = 0.62). The proportion of HCV viremia among index patients was 37%. Of 73 HCWs exposed to HCV RNA from index patients, nine (12.3%; 95%CI, 5.8–22.1%) presented transient viremia, the majority of which occurred within the first two weeks after exposure. None of the workers presented seroconversion or elevation of ALT.

**Conclusions/Significance:**

HCWs of a general University hospital in Cairo were exposed to a highly viremic patient population. They experienced frequent occupational blood exposures, particularly in early stages of training. These exposures resulted in transient viremic episodes without established infection. These findings call for further investigation of potential immune protection against HCV persistence in this high risk group.

## Introduction

Healthcare workers (HCWs) are at increased risk of acquiring blood-borne pathogens, particularly hepatitis B (HBV), hepatitis C (HCV) and human immunodeficiency (HIV) viruses, due to their occupations involving repeated contact with infected patients or contaminated injection material [1].

Reported rates of HCV transmission worldwide range between 0% and 10% for HCWs exposed to blood from HCV RNA positive index patients via needle prick injury, with a point estimate of 0.5% (95% CI; 0.39–0.65%) [2]. The main determinants of transmission in this population are the use of a hollow-bore needle, severity of injury, and the viral load of the index patient [3]. Prior studies into the existence of transient HCV viremia have documented cases among individuals with high-risk exposures, such as HCWs [4] or prison inmates [5], [6]. Additionally, some studies have shown that subjects with high-risk exposure to HCV could induce cell-mediated immunity in the absence of detectable viremia or seroconversion, among HCWs [7], [8], people living with HCV-infected subjects [9], [10] and sexual partners of infected individuals [11], [12].

In Egypt, where HCV prevalence is the highest in the world, 14.7% of the population of 15–59 years present anti-HCV antibodies (Egypt Demographic and Health Survey, 2008) [13]. The origin of this epidemic has been largely attributed to the mass parenteral antischistosomal treatment from the 1960 s to the early 1980 s, which employed insufficiently sterilized injection equipment [14]. As in other developing countries, infection control guidelines, safety measures and basic disposable supplies are not available in all hospital settings [15], [16], [17]. In addition, overuse of injections may lead to an increased risk of exposure to needle stick injuries and to disease [18], [19], [20]. Talaat et al. estimated that the annual number of needle stick injuries was 4.9 per HCW per year [21]. HCWs are therefore at a particularly high risk of acquiring HCV infection. The aim of the research was to study the risk and characteristics of HCV transmission following occupational blood exposure (OBE) in Cairo. Specifically, we characterised OBE at a University hospital and assessed HCV viremia and seroconversion following OBE among HCWs.

## Methods

### Ethics Statement

The study protocol received approval from the Ethics Committee of the National Hepatology and Tropical Medicine Research Institute (NHTMRI) in Cairo and from the Committee for Biomedical Research (CoRC) at Institut Pasteur Paris. At screening, HCWs and index patients signed an informed consent form. Enrolled HCWs signed an additional consent form.

### Conduct of the Study

The study took place in 2008–2010 at Ain Shams Hospital, a University hospital located in Cairo, Egypt, with over 3 200 beds and 4 500 employees. As part of a prick injury control program initiated in 2004, HCWs were encouraged to immediately report to one of the prick injury clinics following possible exposure to patient blood or fluid.

All HCWs currently employed at the internal medicine, obstetrics/gynaecology, surgery and paediatrics wards who reported an episode of OBE were eligible for the screening visit. Anti-HCV negative HCWs were then enrolled and followed up in a prospective 6-month cohort if the enrolment occurred within seven days of the OBE and if a blood sample from the index patient was HCV RNA positive. In accordance with the Centers for Disease Control and Prevention, occupational blood exposure was defined as a percutaneous injury (e.g., a needle stick or cut with a sharp object) or contact of mucous membrane or non-intact skin (e.g., exposed skin that is chapped, abraded, or afflicted with dermatitis) with blood, tissue, or other body fluids that are potentially infectious.

At the screening visit, HCWs answered a brief questionnaire and a 5 ml blood sample was collected by a nurse and tested for HCV antibody (anti-HCV), HCV RNA and Alanine aminotransferase (ALT). If the index patient could be reached, a 5 ml blood sample was also collected and tested for anti-HCV and HCV RNA. At the enrolment visit (week 0), which took place 1–2 days after screening, HCWs were administered a questionnaire on sociodemographics, history of and current OBE incident (e.g. exposure type, severity of the injury, protective measures) and a blood sample was collected. Workers and patients found to be already infected with HCV were referred for further medical care under the national program at the National Hepatology and Tropical Medicine Research Institute (NHTMRI) in Cairo. During follow-up visits scheduled at 2, 4, 8, 12 and 24 weeks following the OBE, HCWs were administered a questionnaire regarding any further occurrence of OBE or risk exposures since the last visit and a blood sample was taken for anti-HCV, HCV RNA and ALT testing. HCWs were called by monitors prior to their scheduled visits and called back if they missed a visit.

### Biological Materials

Screening samples that were positive for anti-HCV at the Emergency Laboratory at Ain Shams Specialized Hospital using Enzyme Immunosorbent Assay (EIA) were tested again at the Viral Hepatitis Research Laboratory (VHRL) (NHTMRI, Cairo) for confirmation using AxSYM HCV, Version 3.0, based on chemoluminescence assay, Abbott Laboratories, according to the manufacturer’s instructions. Those positive by the two serological tests were considered positive. All samples from HCWs and patients were also tested for HCV RNA by use of a qualitative nested reverse transcriptase-polymerase chain reaction (RT- PCR) assay with a detection limit of 50 IU/ml (VHRL ‘in house’ assay) [22]. HCV RNA positive index patient samples were tested with real-time PCR (Abbott RealTime® HCV kit, detection limit 12 IU/mL) at the VHRL to determine viral load. All remaining aliquots were frozen and stored at −80°C. Liver function testing (ALT) was performed using routine tests.

Genotyping and viral load determination of positive worker/patient samples were performed at the virology laboratory at Toulouse Purpan Hospital, France. HCW and index patients’ positive HCV RNA samples were sequenced in the NS5B region of the viral genome to determine the viral strain.

### Statistical Methods

#### Sample size

Based on the previous implementation of the prick injury clinic program at Ain Shams, 400 HCWs were expected to report an OBE to the clinic during a two-year inclusion duration. Of these HCWs, we hypothesised that approximately 90% would not be included in the cohort study (HCV RNA negative index patient, anti-HCV positive HCW at screening, prick injury not involving an index patient and refusal to participate). Thus, the expected number of HCWs at risk for HCV transmission after OBE was approximately 40, which was considered sufficient to observe several episodes of viremia.

#### Data analysis

Data entry was performed using Access® 2003 software (Microsoft Corporation, Redmond, WA, USA). Analyses were performed using Stata® 11.0 (StataCorp LP, Texas, USA). At baseline, crude and age-standardized anti-HCV prevalence rates and their 95% exact confidence intervals (95% CI) were estimated among screened HCWs, using the age distribution of the population of Cairo governorate (2008 Demographic and Health Survey, DHS) as the standard. Logistic regression analysis was used to study risk factors associated with HCV seropositivity of HCWs at screening. Variables with p values<0.25 in the univariate analysis were entered with a stepwise backward procedure in a multivariate model and kept in final model if p<0.05.

In the cohort, the cumulative incidence of viremia among HCWs exposed to HCV RNA was calculated (percentage and 95% CI). Transient viremia was defined as any detection of HCV RNA in serum at any visit (except screening), followed by non-detection of HCV RNA in serum for the remainder of the study. HCWs developing transient viremia were compared with HCWs who did not, using Mann-Whitney and Chi^2^ Fisher exact tests for quantitative and categorical variables, respectively.

## Results

### Description of the Study Population

**Figure 1:**
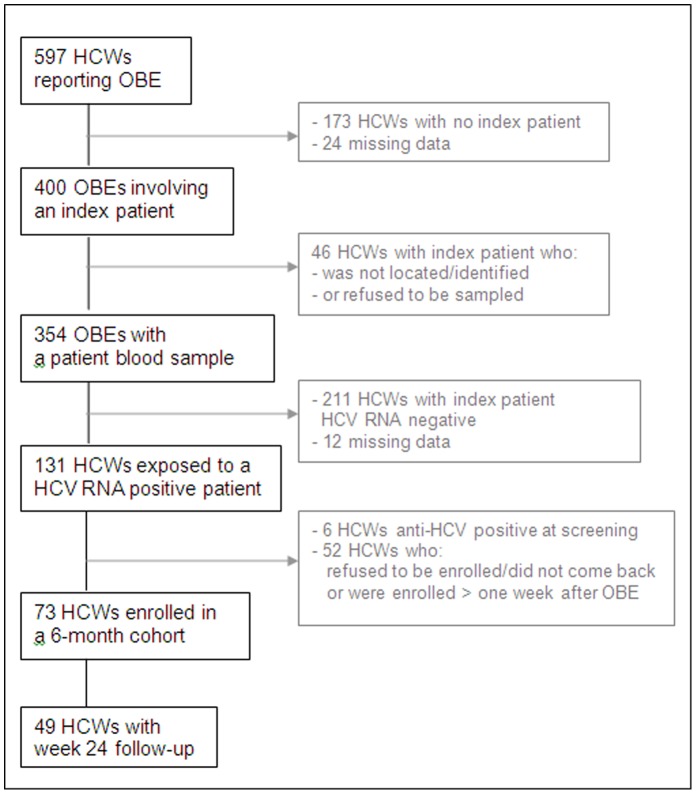
Study flowchart, Ain shams University Hospital, Cairo. HCW: healthcare worker; OBE: occupational blood exposure, IP: index patient; HCV-Ab: Hepatitis C virus antibody.

**Figure 2:**
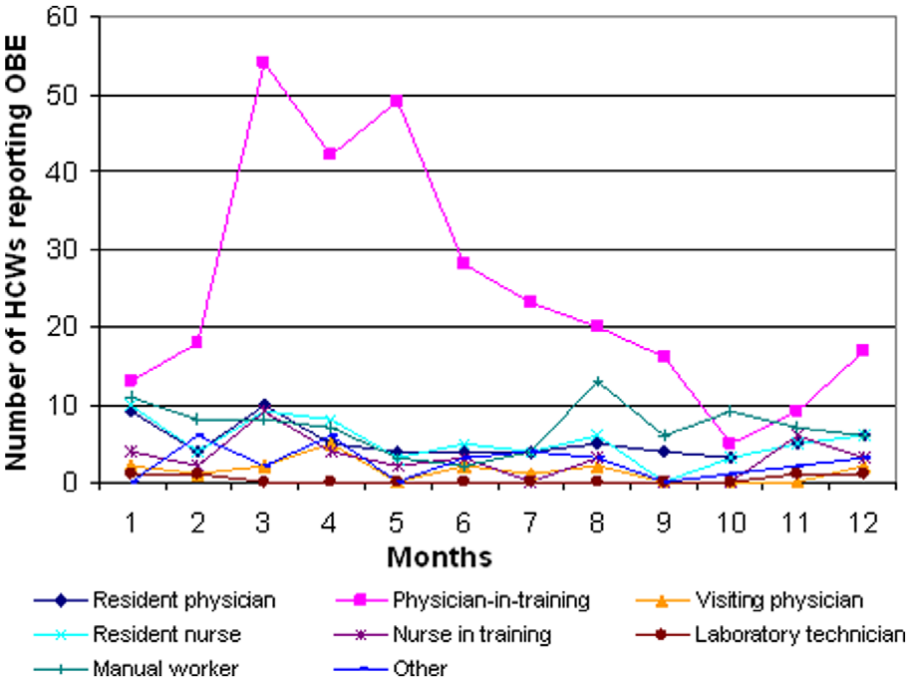
Reported OBEs according to month and occupation, 2008–2010, Ain shams University Hospital, Cairo. OBE: occupational blood exposure; HCW: healthcare worker.

Overall, 597 HCWs reported an OBE from August 2008 to September 2010 (Figure 1). Among these HCWs, 294 (49.3%) were physicians-in-training (i.e. in first year of residency) (Table 1). There were 219 HCWs (36.7%) in the internal medicine department and 146 (24.4%) in the surgery department. Their median age was 24 years [Q_25–75_, 24–28]. The median delay between exposure and reporting was one day [Q_25–75_, 0–2]. A peak of OBE was observed for physicians-in-training between March and May (Figure 2). This three-month period represented 50% of all OBE for physicians in training versus 30% of all OBE for other staff (p<0.001).

**Table 1:** Baseline characteristics of the 597 healthcare workers and factors associated with anti-HCV prevalence at screening, Ain shams University Hospital, Cairo.

Characteristics	HCWs (n = 597) No. (%)	Anti-HCV positive (n = 43) No. (%)	Crude OR [95% CI]	P value (Chi^2^ test)
**Gender**				
Women	297 (49.8)	16 (5.4)	1	
Men	300 (50.2)	27 (9.1)	1.74 [0.92–3.31]	0.086
**Age group**				
<24 years	142 (23.8)	5 (3.5)	1	
24–30 years	331 (55.4)	15 (4.5)	1.30 [0.46–3.65]	<0.001
>30 years	121 (20.3)	23 (19.0)	6.52 [2.39–17.75]	
Missing	3 (0.5)			
**Place of residence**				
Cairo governorate	417 (69.9)	27 (6.5)	1	
Other	166 (27.8)	16 (9.6)	1.54 [0.80–2.93]	0.19
Missing	14 (2.3)			
**Job position**				
Physician-in-training^a^	294 *(*49.3)	8 (2.7)	1	
Resident physician	63 (10.6)	5 (7.9)	3.13 [0.99–9.90]	
Resident nurse	63 (10.6)	7 (11.1)	4.53 [1.58–13.02]	
Nurse in training	36 (6.0)	2 (5.6)	2.10 [0.43–10.27]	<0.001
Manual worker	85 (14.2)	17 (20.0)	9.18 [3.80–22.17]	
Visiting physician	17 (2.8)	3 (17.7)	7.63 [1.82–31.94]	
Other	36 (6.0)	1 (2.8)	1.02 [0.12–8.38]	
Missing	3 (0.5)			
**Department**				
Internal medicine	219 (36.7)	8 (3.7)	1	
Surgery	146 (24.4)	15 (10.3)	3.08 [1.27–7.46]	
Obstetrics/Gynaecology	131 (21.9)	8 (6.1)	1.71 [0.62–4.66]	0.02
Paediatrics	32 (5.4)	3 (9.4)	2.72 [0.68–10.82]	
Other	47 (7.9)	7 (14.9)	4.71 [1.62–13.74]	
Missing	22 (3.7)	2 (9.1)		

OR: Odds-Ratio; 95% CI: 95% confidence interval; HCV: Hepatitis C Virus.

a Physicians-in-training are medical students in first year of residency.

Among the 597 HCWs, 354 (59.3%) had an index patient who could be identified and for whom a blood sample was obtained and 173 (29.0%) had injuries with used injecting materials in trash or containers for which an index patient could not be identified. The mean age (range) of index patients was 45.2 years (1–85).

### HCV Infection Prevalence at Screening

Among the 597 HCWs, 43 (7.2%; 95%CI, 5.3–10%) were anti-HCV positive at screening (27/297 men and 16/295 women, p = 0.09), 549 (92.0%) were anti-HCV negative, 3 (0.5%) had equivocal results and 2 had missing results (0.3%). Systematic HCV RNA testing revealed that 51.2% (22/43) of the anti-HCV positive and 3.8% (21/549) of the anti-HCV negative HCWs were HCV RNA positive.

Factors associated with anti-HCV prevalence of HCWs at screening were age, occupation and department of work (Table 1, univariate analysis). After multivariate logistic regression, only age of HCWs was independently associated with anti-HCV (OR = 6.5, 95%CI 2.4–17.7, for age>30 versus age <24 years). Using the 2008 Demographic and Health Survey age distribution of Cairo as the standard, age-standardized anti-HCV prevalence of HCWs was 11.6% (95%CI, 7.9–15.2%). It was similar to the prevalence in the population of Cairo governorate (10.4%, 95%CI 8.1–12.8%, p = 0.62, Table 2).

**Table 2:** Age-standardized anti-HCV prevalence among healthcare workers reporting an occupational blood exposure, Ain shams University Hospital, Cairo.

	Study (n = 597) %	DHS Cairo^a^ (n = 612) %	P value
Age distribution			
<24 years	23.8%	31.3%	
24–30 years	55.4%	19.3%	<0.001
>30 years	20.3%	49.4%	
Crude anti-HCV prevalence in % (95% CI)	7.2 (5.3–9.6)	10.4 (8.1–12.8)	–
Age-standardized anti-HCV prevalence in % (95% CI)	**11.6 (7.9–15.2)**	Reference **10.4 (8.1–12.8)**	0.62

95% CI : 95% confidence interval; DHS : Demographic and Health Survey.

Age distribution of Cairo governorate population was used as the standard (Egyptian DHS, 2008).

a Cairo age distribution and anti-HCV prevalence are weighed estimates according to DHS parameters.

Among the 354 index patients, 149 (42.1%) were anti-HCV positive, 198 (55.9%) were anti-HCV negative, 4 (1.1%) had equivocal results and 3 had missing data (0.9%). The proportion of anti-HCV positive patients varied according to the department of hospitalization, from 25.0% (5/20) in paediatrics to 55.6% (75/135) in internal medicine. Of all index patients, 131 (37.0%) were viremic, including 122 (81.9%) of the 149 anti-HCV positive patients and 8 (4.0%) of the 198 anti-HCV negative patients (1 viremic patient had missing anti-HCV data).

### Occupational Blood Exposure

Of the 131 HCWs exposed to HCV RNA positive index patients, 73 were enrolled in the prospective study (see Figure 1 for reasons for not participating). Of these subjects, most (78%) reported the OBE within one day. The mean age (range) of HCWs was 24.8 years (16–42). Forty-four (60.3%) were physicians-in-training and 45 (61.6%) worked in the internal medicine department (Table 3). The majority of HCWs were injured using a hollow-bore needle (n = 60, 82.2%). At the time of exposure, 27 HCWs (37.0%) were drawing blood. Forty-one (56.2%) HCWs wore gloves and either immediately washed the affected area after exposure, or used a disinfectant. The mean HCV RNA concentration of index patients was 4.8 log IU/ml, ranging from 2.1 to 7.2 log IU/ml, with a median viral load of 4.9 log IU/ml.

**Table 3:** Characteristics of occupational blood exposure for HCWs anti-HCV negative at screening and exposed to a HCV RNA positive patient, Ain shams University Hospital, Cairo.

Variables	HCWs exposed to HCV RNA and enrolled in study (n = 73) No. (%)
***Sociodemographic characteristics***	
** Gender**	
Women	38 (52.0)
Men	35 (48.0)
** Age**	
<24 years	14 (19.2)
≥24 years	59 (80.8)
**Place of residence**	
Cairo governorate	49 (67.1)
Other	22 (30.1)
Missing	2 (2.8)
**Job position**	
Physician-in-training	44 (60.3)
Resident physician	12 (16.4)
Resident nurse	7 (9.6)
Nurse in training	3 (4.1)
Other	7 (9.6)
**Department**	
Internal medicine	45 (61.6)
Surgery	14 (19.2)
Obstetrics/Gynaecology	9 (12.3)
Other	5 (6.9)
***Occupational exposure***	
**Type of injury**	
Needle prick or sharp object	63 (86.3)
Body fluid splash	1 (1.4)
Missing	9 (12.3)
**If needle, type** a **:**	
Hollow-bore needle	60 (95.2)
Suture needle	2 (3.2)
Other	1 (1.6)
**Exposition to any blood during incident**	
No	43 (58.9)
Yes	19 (26.0)
Do not know	2 (2.8)
Missing	9 (12.3)
**Did the HCW bleed?**	
No	42 (57.5)
Yes	20 (27.4)
Do not know	2 (2.8)
Missing	9 (12.3)
**Exposure while working in the ER**	
No	20 (27.4)
Yes	44 (60.3)
Missing	9 (12.3)
**Task during exposure**	
Drawing blood	27 (37.0)
IV injection	10 (13.7)
Intra-arterial catheter	2 (2.7)
Canula insertion or removal	7 (9.6)
Surgery	7 (9.6)
Lab testing or procedures	3 (4.1)
Trash removal	1 (1.3)
Other	8 (11.0)
Missing	8 (11.0)
**Index patient viral load (HCV RNA)**	
<4.9 log IU/ml (median)	33 (45.2)
>4.9 log IU/ml	35 (48.0)
Missing	5 (6.8)
***Protective measures***	
**Wearing gloves**	
No	21 (28.8)
Yes	43 (58.9)
Missing	9 (12.3)
**Wearing goggles**	
No	60 (82.2)
Yes	3 (4.1)
Missing	10 (13.7)
**Immediate washing**	
No	11 (15.1)
Yes	53 (72.6)
Missing	9 (12.3)
**Use of disinfectant**	
No	19 (26.0)
Yes	45 (61.7)
Missing	9 (12.3)

HCW: healthcare worker; HCV: hepatitis C virus; ER: Emergency Room; IV: intravenous.

a Among HCWs with needle prick injury (n = 63).

### HCV Transmission from Index Patient to Healthcare Worker

Among the 73 HCWs enrolled in the prospective study, 49 (67.1%) reached week 24 follow-up and 61 (83.6%) completed at least 3 follow-up visits. None of the 73 HCWs seroconverted or had elevation of ALT during follow-up. Nine had evidence of transient viremia at one time point during follow-up, representing a cumulative incidence of 12.3% (95%CI, 5.8%–22.1%). Most (66.7%) HCWs had their viremic episode within 2 weeks after exposure. Seven out of the nine viremic HCWs had at least three follow-up visits after their transient viremia, with measurement of anti-HCV and HCV-RNA (all negative).

Viral load ranged from 1.6 to 4.5 log IU/mL for viremic workers with available result (n = 3) and from 3.5 to 5.8 log IU/mL for corresponding index patients (Table 4). Viral load could not be obtained from the remaining viremic HCWs (n = 6), because of low volume available for quantification.

**Table 4:** Characteristics of viremic HCWs following OBE, Ain shams University Hospital, Cairo.

Viremic HCW	Duration of follow-up	Sex	Age (yrs)	Occupation	Department of work	Wearinggloves	Use ofdisinfectant	Delay OBE-viremia (days)	Index patient genotype	Index patient viral load (logIU/ml)	
1	24 W	M	27	Resident physician	GynaecologyObstetrics	Yes	Yes	16	4a	5.79	
2	24 W	M	23	Physician-in-training	Internal medicine	No	No	9	not determined	3.54	
3	12 W	M	25	Resident physician	Internal medicine	–	–	19	4a	5.66	
4	24 W	F	16	Other	Internal medicine	Yes	Yes	3	not determined	4.98	
5	24 W	F	24	Physician-in-training	Internal medicine	Yes	No	14	4n	5.45	
6	24 W	F	17	Student	Internal medicine	No	Yes	7	4a	4.85	
7	8 W	F	23	Physician- in-training	Internal medicine	Yes	No	3	4a	4.52	
8	24 W	M	25	Physician-in-training	Internal medicine	Yes	No	80^a^	4a	5.84	
9	24 W	M	29	Nurse in training	Surgery	No	No	104^a^	not determined	4.91	

HCW: healthcare worker; OBE: occupational blood exposure; M: male, F: female; W: weeks.

a Mid-point between last visit with negative result and visit with positive HCV RNA.

The cumulative incidence of transient viremia was not significantly different between men and women. However, it was different according to the HCW’s age, with 35.7% (5/14) viremic HCWs among those aged below 24 years versus 6.8% (4/59) among HCWs aged 24 or above (p = 0.01). Also, the use of disinfectant after OBE was lower among HCWs who developed viremia than among those who did not (6.7% (3/45) and 26.3% (5/19), respectively; p = 0.04). Mean viral load of index patients did not differ significantly between viremic and non viremic HCWs (5.1 and 4.8 logIU/ml, respectively, p = 0.36).

## Discussion

In this study, we examined HCV exposure among HCWs in Egypt, where the HCV reservoir is considerable. We reported the age-standardized anti-HCV prevalence in HCWs and documented occurrence of transient viremia without seroconversion after a well-characterised occupational blood exposure.

### A Highly HCV Viremic Setting

The first important finding of this study is the extremely high (37%) proportion of patients with HCV viremia among inpatients of a general hospital in Cairo. This is likely explained by the median age of the patients (48 years), thus belonging to the age group known for having the highest HCV prevalence in Egypt [13]; by the possibility that some patients may have been hospitalised for HCV-related complications; and by the high risk of HCV iatrogenic transmission among regularly hospitalised patients. Concurrent with this finding is the high (4%) proportion of patients with positive HCV RNA among those with negative HCV antibodies, suggesting that the majority of those patients might have been recently infected with HCV and are still in the “window period” of their acute infection.

With such a high HCV circulation in the hospital, we expected to find a high HCV prevalence among exposed HCWs, particularly among those of this study reporting OBEs. Surprisingly, the age-standardized anti-HCV prevalence in HCWs was not different from the general population of Cairo (11.6% versus 10.4%, p = 0.62), suggesting that this high exposure to HCV did not translate into a higher rate of infection among HCWs. What we observed instead was a high (12%) rate of transient viremia without seroconversion. This rate may be an underestimation due to irregular and incomplete follow-up, since the detection of the short viremic episodes was dependent on the frequency of testing. Two studies among prison inmates have previously documented transient viremia without seroconversion, but reported longer durations of viremia. In the study among 117 Australian prison inmates, 4 cases of viremia occurred followed by HCV RNA clearance after 2–6 months, and were associated with specific cellular immunity in the absence of seroconversion [5]. In another cohort followed in Germany among 71 HCV RNA positive prisoners, 7 cleared the virus after 8–31 weeks, 4 of whom had continuous anti-HCV negativity [6].

Other studies with systematic investigation among HCWs are limited but are notable as having focused on cellular immune responses. In Germany, ten healthcare workers were followed prospectively after injury with HCV-contaminated needle [7]. None of them seroconverted or had detectable viremia, however HCV specific CD4+ T-cells responses were detected in 4 of the 10 HCWs. In Egypt, in a cross-sectional study among HCWs [23], 29 (55.8%) of 52 seronegative aviremic HCWs had a positive HCV-specific IFN-γ response detected by ELISPOT assay, compared to none of the 15 healthy controls [8]. Studies conducted in individuals living with infected subjects [9], [10], [11], [12] have shown similar findings. These studies highlight the presence of cellular immune responses which may prevent HCV infection and facilitate viral clearance. Injecting drug users (IDUs) are also frequently exposed to HCV and several cohort studies investigating natural history of acute infection and reinfection have been carried out in this population. A recent review by Grebely et al. [24] showed that results are conflicting between studies, with studies reporting a lower incidence of HCV infection in individuals with previous HCV clearance versus those who had not been infected previously and other suggesting that previous spontaneous clearance may not reduce the risk of reinfection. However, as underlined by Grebely, differences in methodology with different HCV RNA testing intervals between studies and limited (or absence of) adjustment on risk behaviour between individuals who cleared a previous infection and those never infected may explain conflicting results. But even though previous clearance of HCV infection may not provide sterilising immunity against reinfection, protection against HCV persistence may be developed in some individuals.

Interestingly, IDUs often are a young population, as our HCWs. However, IDUs rarely have a precisely known date of infection, and they are usually exposed to a more invasive procedure (clear IV injection). In our study, we may hypothesize that the lack of viremia persistence could be linked to immune protection induced by multiple mild exposures. Unfortunately, we were not able to sequence the viruses of HCWs due to the relatively low viral loads, thus preventing us from definitely linking these transient viremic episodes to the OBE. Having low viral load levels among HCWs is nevertheless consistent with the transient nature of the phenomenon and the timing of OBE that was documented.

Finally, at the initial screening, the proportion of HCV RNA positivity among anti-HCV positive HCWs increased with age (6/20 = 30% vs. 16/23 = 69.6%, among those ≤30 years and >30 years, respectively, p = 0.01). Among our younger HCWs, we can assume that they were infected at a young age; however for older HCWs, it is difficult to disentangle the effect of age at infection from the effect of multi-exposure on subsequent HCV chronicity. Age could also be associated with a decrease in immunity, with the T cell repertoire potentially exhausting over time after multiple stimulations, leading to a higher susceptibility at older age. By comparing our HCWs to the population of Cairo, and when considering subjects ≤30 years only, the rate of chronic HCV infections among anti-HCV positive HCWs was significantly lower than in Cairo population (6/20 = 30% vs 174/265 = 65.7%, respectively). We believe that at equal young age, our health workers may be more multi-exposed than the general population, thus partly explaining their lower HCV-RNA positivity.

### Occupational Blood Exposure

Physicians-in-training were the highest group to report OBE. A study conducted among HCWs in a German University hospital showed that medical students reported more OBEs than other employees (nurses and medical doctors) [25]. In 2008, a survey among medical residents in France [26] indicated that most OBEs occurred during the first year of residency. We also showed the seasonality of OBEs among physicians-in-training (Figure 2), most of the OBEs taking place in the spring, at the time HCWs start working in a new ward, as shown in other studies [27]. Manual workers also represented a high proportion of reported OBEs (14.3%) although only one was enrolled in our follow-up study, most of them getting injured while removing trash and thus having no identifiable index patient. A large proportion of these HCWs were working in the surgery department, similar to an Italian prospective study [28].

In a case-control study among European HCWs [3], risk factors for HCV transmission (seroconversion) after OBE included deep injuries and procedures involving hollow-bore needle placement in the source patient’s vein or artery. In our study, almost all HCWs had been injured with a hollow-bore needle. With 9 cases of transient viremia, we were unable to identify known risk factors for HCV transmission. Of interest, the use of disinfectant after OBE was associated with a lower risk of subsequent transient viremia.

### Conclusions

We have shown that, despite being in a setting with high HCV infection prevalence (37% of inpatients being HCV viremic), HCWs had similar baseline anti-HCV prevalence as compared to the population of Greater Cairo. Instead, HCWs had frequent transient viremia after OBE involving a HCV RNA positive index patient (12%), but no HCW seroconverted until 24 weeks after OBE. Cellular and innate immune responses might be associated with this apparent protection against HCV persistence in this high-risk population and should be further investigated in order to identify specific immunologic profiles related to HCV clearance. In this context of large HCV reservoir, OBEs were frequently reported. Infection control needs to be improved, and more resources should be allocated to safety measures. In particular, young HCWs and manual workers need to be sensitized to the risk of HCV infection and the application of standard hygiene procedures.
